# Lessons from protozoans: Phosphate sensing and polyphosphate storage in fungi

**DOI:** 10.1371/journal.ppat.1010298

**Published:** 2022-03-03

**Authors:** Taissa Vila, Susana Frases, Fabio M. Gomes

**Affiliations:** 1 Laboratório de Biologia Celular de Fungos–Instituto de Biofísica Carlos Chagas Filho–Universidade Federal do Rio de Janeiro, Rio de Janeiro, Brazil; 2 Laboratório de Biofísica de Fungos–Instituto de Biofísica Carlos Chagas Filho–Universidade Federal do Rio de Janeiro, Rio de Janeiro, Brazil; 3 Laboratório de Ultraestrutura Celular Hertha Meyer–Instituto de Biofísica Carlos Chagas Filho–Universidade Federal do Rio de Janeiro, Rio de Janeiro, Brazil; University of Maryland, Baltimore, UNITED STATES

## Phosphate is a prebiotic component and an essential component of a living cell

In order to function, cells must be able to uptake, store, and mobilize nutrients according to the availability of their surrounding environment. Opposite to most heterotrophic organisms, fungi secrete enzymes to the environment that reduce nutrient complexity before uptake and metabolization. Despite this remarkable difference, yeasts such as *Saccharomyces cerevisiae* have been extensively used as a model to study the biochemical and molecular regulatory pathways that control how eukaryotic cells respond to the environment. In that sense, how fungi coordinate the supply and mobilization of nitrogen and carbon for the synthesis of biomolecules, such as amino acids and sugars, has been the focus of intensive research [1,2]. In contrast, the coordination of phosphate (Pi) homeostasis has attracted less attention from the scientific community.

Pi is thought to have a central role in prebiotic chemistry (before the evolution of cells), and to be a key component for the evolution of life, as previously reviewed [3]. In modern cells, Pi is found in the structure of several macromolecules where it helps to coordinate cellular biochemistry and metabolism. For example, as phospholipids, it is incorporated into the cell membranes, defining the boundaries between the intracellular and extracellular space; as nucleoside phosphates, it provides free energy potential for chemical reactions to develop; as nucleotides, it allows the flow of genetic information. Thus, Pi levels must be tightly regulated inside the cells. Not surprisingly, Pi sensing is interconnected with other nutrient-sensing pathways in yeasts [4,5]. More recently, comparative studies of phosphate homeostasis in the pathogenic yeasts *Candida albicans* [6] and *Cryptococcus neoformans* [7] have provided interesting insights on fungi nutrient sensing and virulence that deserve further investigation.

## Fungi have a unique pathway to sense and regulate intracellular phosphate levels

**Fig 1:**
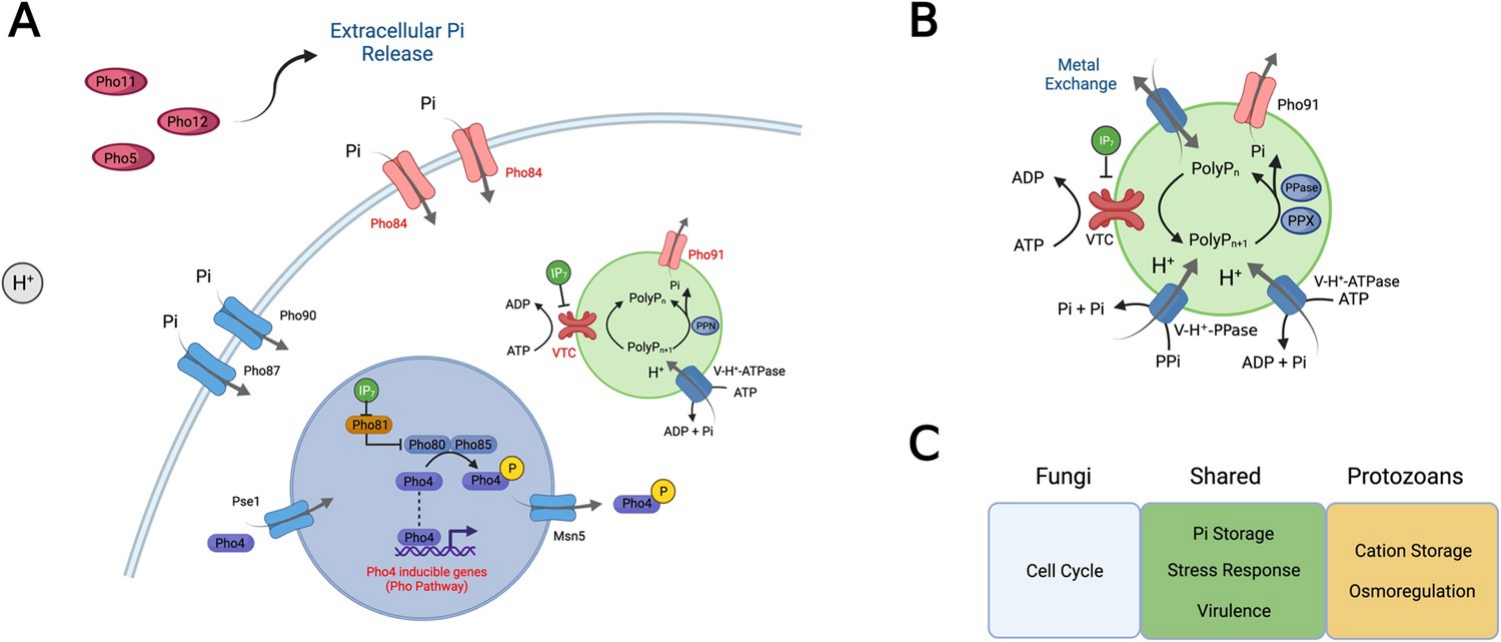
Pi homeostasis in unicellular eukaryotes shares conserved features and roles. (A) In fungi, a Pi sensing system has been described that coordinates Pi mobilization from the environment with Pi uptake and storage as PolyP. When kept at proper Pi levels, the low-affinity Pi transporters Pho87 and Pho90 regulate Pi import from the external medium. Upon reduction of cytoplasmic Pi levels, Pho81 inhibits the Pho4 phosphorylating activity of the cyclin-CDK Pho80-Pho85, preventing the nuclear export of phosphorylated Pho4 by Msn5. Unphosphorylated Pho4, which enters the nuclei by interaction with Pse1, is a transcription factor that drives the expression of several genes involved in Pi mobilization and uptake (in red letters). VTC activation drives vacuolar PolyP synthesis, which acts as a Pi reservoir mobilized by specific polyphosphatases. Levels of InsPi, such as 5-diphosphoinositol pentakisphosphate (IP7), correlate with cellular Pi levels and regulate several proteins involved with Pi homeostasis through binding with their SPX domains. (B) Protozoan acidocalcisomes share several features also found in fungi vacuoles, such as ATP-dependent H^+^-pumps (V-H^+^-ATPase), VTC, and phosphate exporters. Acidocalcisomes pH is also regulated by the presence of a PPi-dependent proton pump (V-H^+^-PPase), which have not been not found in the genome of fungi. Acidocalcisomes also have several cation channels that drive cation homeostasis, which has not been as extensively investigated in fungi models. (C) PolyP and Pi homeostasis collectively have several roles in cellular physiology and disease progression, some of which have been demonstrated in both models. CDK, cyclin-dependent kinase; VTC, vacuolar transporter chaperone complex.

The first evidence for a pathway that regulates Pi uptake and mobilization upon Pi deprivation in *S*. *cerevisiae* was identified around the 1960s [8], and by the mid-1990s, the core of this pathway was already well described [9]. This pathway—known as the PHO pathway—relies on the activation of the Pho4 transcription factor and the expression of several genes that regulate Pi homeostasis. Unphosphorylated Pho4 enters the nuclei by interaction with its import receptor Pse1 [10]. On Pi supplemented conditions, the cyclin-dependent kinase (CDK) complex Pho80-Pho85 phosphorylates Pho4 [11], which is removed from the nuclei by the nuclear export Msn5 [12]. Upon Pi starvation, the CDK inhibitor Pho81 inactivates Pho80-Pho85 [13], allowing unphosphorylated Pho4 to accumulate in the nuclei, driving Pho4-induced gene expression [10] (Fig 1A). In *S*. *cerevisiae*, around 20 genes are regulated by Pho4, including phosphatases (e.g., Pho5, Pho10, Pho12) and high-affinity phosphate transporters (Pho84, Pho89) [14].

As Pi is mobilized in the extracellular space and imported through phosphate transporters, the cells must have a mechanism to control cytoplasmic Pi concentration to avoid toxicity. In that sense, Pi uptake is coordinated with the synthesis of a storage macromolecule called polyphosphate (PolyP) at the yeast vacuole. The polyP is an inorganic polymer of Pi residues condensed by phosphoanidride bonds. Early NMR studies had shown that PolyP stores are mobilized until depletion during Pi starvation to sustain cytoplasmic Pi levels [15], and the vacuolar Pi transporter Pho91 was associated with Pi export from the vacuole to the cytoplasm [16]. In unicellular eukaryotes, the PolyP synthesizing machinery remained undefined for decades, but it was later shown that the vacuolar transporter chaperone (VTC) complex catalyzes the synthesis of PolyP from ATP [17]. Accordingly, VTC is under the regulation of Pho4 [14], providing a link between Pi uptake and vacuolar PolyP synthesis.

Recently, exciting findings have shed light on the roles of inositol polyphosphates (InsP) in the coordination of Pi homeostasis. InsP was shown to regulate several proteins involved in Pi homeostasis, including VTC and Pi transporters, by interacting with their SPX domain [18]. As InsP levels are known to oscillate depending on Pi availability [19], a role of InsP in sensing Pi levels and transducing Pi availability by regulating Pi homeostasis-linked proteins has been proposed [18].

## Pi levels and cell growth must be tightly coordinated with other nutrient-sensing pathways

As expected, a crosstalk between the Pho and other nutrient-sensing pathways has been suggested. Pi availability has been shown to modulate TOR signaling through TORC1, a pathway originally designated to sense nitrogen levels inside the cells [4]. Furthermore, the crosstalk between PHO, PKA, and TOR pathways was proposed to regulate NAD+ metabolism [20]. Tor1 kinase was also shown to downmodulate Pho84 expression upon restoration of Pi levels (and TOR activation). According to their role as a Pi reservoir, *S*. *cerevisiae* mutants lacking PolyP were shown to accumulate less dNTPs and had their cell cycle impaired in phosphate-limiting conditions [21].

## The Pho pathway is an essential component for the virulence of fungi

In *C*. *albicans*, Pho4 was found to regulate a much larger number of genes than in *S*. *cerevisiae* [22]. Whether this reflects the complexity of the adaptation between host and environment remains to be elucidated. Further studies on these models revealed that the Pho pathway is essential for stress tolerance and virulence [22,23]. This is not necessarily surprising as invading cells must adapt to a dynamic environment and multiple sources of stress throughout infection. More specifically, *Pho4* null mutants have increased sensitivity to osmotic, cationic, oxidative, and alkaline stress. Interestingly, while phosphate starvation and alkaline stress resulted in Pho4 accumulation in the nuclei, and thereby activation of the Pho Pathway, cationic and superoxide stress did not correlate with Pho4 nuclear localization [22]. Overall, this might suggest an alternative mechanism by which Pho4 responds to different stressors.

Accordingly, Pi homeostasis was shown to be essential for virulence in a selection of 43 isolates representing the major *C*. *albicans* clades [24]. In an additional study, *C*. *albicans* clinical isolates from the stool of critically ill patients have shown enhanced virulence and filamentation under Pi depletion [25]. While these findings were not observed in other laboratory prototype strains, such as SC5314 and SN152, they might reflect exacerbated features of a more conserved role of Pi sensing in virulence. Similarly, ablation of Pi transporters resulted in reduced formation of *C*. *neoformans* capsule and melanin [26], two key virulence factors. In *C*. *neorformans*, Pho4 virulence was reduced in vivo and correlated with a reduction in growth under the alkaline conditions of the blood and brain dissemination [27].

So far, there is no clear and single explanation on the mechanism involved in Pho-induced stress resilience and virulence. Pho4 was shown to be important for *C*. *albicans* virulence against *Caenorhabditis elegans* in phosphate-rich media [22], indicating that Pho4 might also be important to coordinate the response against multiple stressors apart from Pi depletion. Interestingly, PolyP has been considered a key determinant of osmotic, cationic, and oxidative stress resistance in several fungal and nonfungal models, as previously reviewed [28]. Additionally, PolyP was shown to be essential for cell cycle progression [21]. It is then tempting to speculate that Pho4-induced PolyP synthesis by the VTC complex contributes to stress resilience and, as a consequence, virulence and host adaptation.

## Other pathogens have striking similarities in how they store PolyP and its importance for virulence

In protozoans, PolyP is mainly stored in organelles known as acidocalcisomes. While several regulators of the PHO pathway are not found in protozoans, acidocalcisomes share remarkable molecular similarities with yeast vacuoles, including acidification mediated by the presence of V-H^+^-ATPases, a distinct elemental composition derived from the storage of high levels of metals, and PolyP accumulation [29] (Fig 1B). The later identification of VTC as the key mediator of PolyP synthesis in fungi vacuoles and protozoan acidocalcisomes comprises one more similarity between these 2 models. Further studies using *vtc* mutants confirmed that PolyP storage in acidocalcisomes is important for the virulence and/or survival under infection conditions of trypanosomatid and apicomplexan protozoans [30–33]. More recently, a conserved role for InsP in regulating Pi homeostasis by interaction with SPX domains has also emerged in *Trypanosoma brucei* [34].

While the molecular machinery of Pi homeostasis has been better described in fungi models, the cell biology of acidocalcisomes has been more extensively studied. Thus, protozoan models might provide interesting insights on how yeast vacuoles coordinate Pi homeostasis and its role in other cellular functions. For example, X-ray elemental analysis has been extensively used for tracking acidocalcisome content and was coupled with fluorimetric studies to highlight the role of organelle acidification in PolyP synthesis, and the tight coordination between PolyP mobilization and other acidocalcisome stored ions homeostasis [35]. Acidocalcisomes have been shown to interact with several subcellular compartments and to mobilize their luminal content, raising the question of whether similar patterns are also to be found in fungi models. Also, contact sites between acidocalcisomes and mitochondria have been identified [36] and a dynamic model of acidocalcisome fusion with other subcellular structures has been proposed to coordinate osmoregulation [29].

## Conclusions

The ability to obtain nutrients from the environment is tightly associated with the development of saprophytic fungi. At the same time, this metabolic plasticity is used in essential survival activities during pathogenic processes. Pi and PolyP metabolism is fundamental for several physiological functions in fungi and protozoans and has been linked with pathogen virulence and disease progression (Fig 1C). In prokaryotes, drugs that block PolyP synthesis—by targeting polyphosphate kinases (PPKs) (the PolyP polymerase enzyme found in bacteria)—have shown promising results against *Mycobacterium tuberculosis* and *Pseudomonas aeruginosa* [37,38]. Despite PHO4 and several genes of the PHO pathway not being conserved between fungi and protozoans, VTC homologs carrying polyphosphate polymerase are present in protozoans. In contrast to the enzymes regulating InsP, VTCs are not found in animals, granting reduced off-target toxicity in human or mammalian cells and increasing its potential as a pharmaceutical target. It is then tempting to speculate that drugs targeting Pi homeostasis and PolyP metabolism could be explored for therapeutic applications. However, null *vtc4* mutants failed to identify hypersensitivity to stressors in *C*. *albicans* [25], and further questions will be needed to address the real impact of PolyP synthesis and mobilization under fungi stress conditions. Here, the identification of conserved features of PolyP metabolism and PolyP storage in fungi and protozoans might shed a light on the key mechanisms coordinating pathogen virulence.
